# Detection of depression in low resource settings: validation of the Patient Health Questionnaire (PHQ-9) and cultural concepts of distress in Nepal

**DOI:** 10.1186/s12888-016-0768-y

**Published:** 2016-03-08

**Authors:** Brandon A. Kohrt, Nagendra P. Luitel, Prakash Acharya, Mark J. D. Jordans

**Affiliations:** Research Department, Transcultural Psychosocial Organization (TPO) Nepal, Baluwatar Kathmandu, G.P.O Box 8974/C.P.C. Box 612, Nepal; Duke Global Health Institute, Department of Psychiatry and Behavioral Sciences, Duke University School of Medicine, Durham, USA; Center for Global Mental Health, Institute of Psychiatry, King’s College London, London, UK; HealthnetTPO, Research Department, Amsterdam, The Netherlands

**Keywords:** Cross-cultural, Cultural concepts of distress, Depression, Global mental health, Low- and middle-income countries, Screening, Validation

## Abstract

**Background:**

Despite recognition of the burden of disease due to mood disorders in low- and middle-income countries, there is a lack of consensus on best practices for detecting depression. Self-report screening tools, such as the Patient Health Questionnaire (PHQ-9), require modification for low literacy populations and to assure cultural and clinical validity. An alternative approach is to employ idioms of distress that are locally salient, but these are not synonymous with psychiatric categories. Therefore, our objectives were to evaluate the validity of the PHQ-9, assess the added value of using idioms of distress, and develop an algorithm for depression detection in primary care.

**Methods:**

We conducted a transcultural translation of the PHQ-9 in Nepal using qualitative methods to achieve semantic, content, technical, and criterion equivalence. Researchers administered the Nepali PHQ-9 to randomly selected patients in a rural primary health care center. Trained psychosocial counselors administered a validated Nepali depression module of the Composite International Diagnostic Interview (CIDI) to validate the Nepali PHQ-9. Patients were also assessed for local idioms of distress including heart-mind problems (Nepali, *manko samasya*).

**Results:**

Among 125 primary care patients, 17 (14 %) were positive for a major depressive episode in the prior 2 weeks based on CIDI administration. With a Nepali PHQ-9 cutoff ≥ 10: sensitivity = 0.94, specificity = 0.80, positive predictive value (PPV) =0.42, negative predictive value (NPV) =0.99, positive likelihood ratio = 4.62, and negative likelihood ratio = 0.07. For heart-mind problems: sensitivity = 0.94, specificity = 0.27, PPV = 0.17, NPV = 0.97. With an algorithm comprising two screening questions (1. presence of heart-mind problems and 2. function impairment due to heart-mind problems) to determine who should receive the full PHQ-9, the number of patients requiring administration of the PHQ-9 could be reduced by 50 %, PHQ-9 false positives would be reduced by 18 %, and 88 % of patients with depression would be correctly identified.

**Conclusion:**

Combining idioms of distress with a transculturally-translated depression screener increases efficiency and maintains accuracy for high levels of detection. The algorithm reduces the time needed for primary healthcare staff to verbally administer the tool for patients with limited literacy. The burden of false positives is comparable to rates in high-income countries and is a limitation for universal primary care screening.

**Electronic supplementary material:**

The online version of this article (doi:10.1186/s12888-016-0768-y) contains supplementary material, which is available to authorized users.

## Background

Major depressive disorder is a major contributor to the global burden of disease and was recently ranked as the second leading cause of years lived with disability worldwide [1, 2]. The greatest projected increase in disability due to depression is in low-and middle-income countries (LMIC) where the availability of mental health services is most limited [3]. The World Health Organization (WHO), United Kingdom’s Wellcome Trust, United States’ National Institute of Mental Health (NIMH), and government development programs such as the United Kingdom’s Department for International Development (DFID) and Grand Challenges Canada (GCC) have supported major initiatives to address the gap between the burden of mental illness and lack of mental healthcare. These initiatives are contributing to an evidence base for the effectiveness of treating depression in primary care and community settings, including treatment by non-specialist health workers [4].

Despite burgeoning initiatives to integrate treatment for depression into primary care in LMIC, there is an increasing realization of the challenges in detecting depression [5, 6]. Under-detection of depression can limit the population-wide impact of increased availability of services. Therefore, approaches to improve detection are needed. One approach is screening, which is defined as “the use of questionnaires concerning the symptoms of depression or small sets of questions about depression to identify patients who may have depression but who have not sought treatment and whose depression has not already been recognized by health care providers,” [7].

There are contrasting perspectives toward the use of screening in high-income countries (HIC) [7]. For example, in the United States and Canada, primary care screening for depression is recommended in the context of adequate available services [8, 9]. The US Preventive Services Task Force recommends universal adult screening with a B grade of evidence, suggesting moderate to high certainty of moderate to substantial benefit. The main risks are in the context of pharmacological treatment following screening are increased risk of suicidal behavior and gastrointestinal bleeding in younger and older adults respectively [9]. In contrast, the United Kingdom’s National Institute for Health and Clinical Excellence (NICE) does not recommend depression screening; instead the guidelines suggest asking about current depression symptoms only when there is a clinical suspicion, a chronic physical health problem with functional impairment, or a history of depression [10].

The contrasting approaches toward screening have arisen from criticisms based on the interacting factors of (a) high rates of false positives, (b) costs associated with screening, and (c) poor quality of routine care received after screening [7]. In HIC settings, approximately four to six patients out of ten are falsely screened positive by ultra-short and short screening tools, such as the Patient Health Questionnaire (PHQ-2 and PHQ-9) [11–13]. This high rate of false positive and potentially inappropriate treatment has contributed to NICE’s recommendation against universal screening [7]. The challenges of screening are magnified in LMIC where instruments have not been validated and relative costs of screening can be greater because of the need for health workers to verbally administer questionnaires among populations with low literacy rates [14]. Approaches are needed to minimize time and human resource burdens as well as maximize accuracy of depression detection in LMIC and other low-resource settings.

The pairing of culturally-salient idioms of distress with culturally and clinically-validated assessment tools could be a solution to improve depression detection in LMIC. Idioms of distress and cultural concepts of distress refer to “ways that cultural groups experience, understand, and communicate suffering, behavioral problems, or troubling thoughts and emotions,” [15]. Assessments of idioms of distress may be briefer than standard tools, more culturally acceptable, and less stigmatizing if the appropriate terms are selected [16]. However, these tend to be nonspecific terms that include a wide range of distress beyond a single psychiatric disorder [17]. In a meta-analysis of 20 depression studies comparing idioms of distress with depression assessments, the sensitivity of idioms of distress to identify depression was 0.61, specificity was 0.78, positive predictive value was 0.41, and negative predictive value was 0.88 [16]. This demonstrates that cultural idioms of distress, used in isolation, miss many depression diagnoses and would not solve the problem of high false positive rates in screening. However, combining local idioms with validated tools may have promise to improve depression in an accurate and feasible manner.

We propose a screening approach that that combines idioms of distress with a transculturally translated and validated instrument. Our goal was to transculturally translate and clinically validate the PHQ-9 while simultaneously evaluating the psychometric properties of an idiom of distress screening item. The validation outcomes would then be used to explore the potential for a combined screening algorithm that could be used to detect depression in primary care with minimal false positives and limited additional burden on health workers conducting the screening.

## Methods

### Setting

Nepal is a low-income country, one of the poorest countries in Asia, and is categorized by the World Bank as a fragile state [18]. The total population of the country is approximately 27 million with the majority (83 %) living in the rural areas [19]. The country is in a transition period following a 10-year intra-state conflict between government forces and Maoists insurgents. The conflict raged from 1996 to 2006 and claimed over 13,000 lives. Previous studies have demonstrated the impact of political violence on psychosocial wellbeing and mental health in Nepal. These studies, conducted after the conclusion of the conflict, have identified high rates of depression ranging from 17–40 % of the general adult population [20, 21]. Depression is associated with impaired functioning [21, 22], and suicide is the leading single cause of death among women of reproductive age in Nepal [23]. This burden of disease is in the context of limited specialized mental health services in Nepal and throughout South Asia [24]. At the time of a 2011 needs assessment, there were fewer than 75 Nepali psychiatrists in clinical practice, with the majority of these working in large urban areas or outside of Nepal in high-income countries [25]. Recently, in 2015, two major earthquakes and a series of more than 400 major aftershocks have raised concern for additional mental health burdens on the population [26].

It is against the backdrop of recent violence and natural disasters, ongoing poverty, high depression burden, and a lack of mental health services that the DFID-sponsored Programme for Improving Mental Health Care (PRIME) was implemented in Nepal [27]. PRIME aims to improve the coverage of treatment for priority mental disorders by implementing and evaluating a comprehensive mental health care package, integrated into primary health care in five LMICs (Nepal, India, South Africa, Ethiopia and Uganda) [28]. The care package includes the provision of psychosocial and pharmacological interventions by non-specialized primary health workers (following the World Health Organization mental health Gap Action Programme (mhGAP)-Intervention Guide [29]) and community counselors [27]. Currently, no mental health services are systematically available in primary care settings [25, 30]. PRIME and the depression detection research described here are implemented by Transcultural Psychosocial Organization (TPO) Nepal, a Nepali non-governmental mental health research and training organization [31]. PRIME is implemented in Chitwan, a district in southern Nepal.

Anthropological, linguistic, and other cultural studies in Nepal have revealed a range of idioms related to depression but no direct one-to-one translation, i.e., there is no Nepali word that captures the concept of depression in a manner synonymous with the English language Western cultural and biomedical construct [32, 33]. In anthropological research in Nepal, we previously documented broader categorical terms for mental illness (*maanasik rog*) and mental problems (*maanasik samasya)* that are typically associated with psychosis and highly stigmatizing. These studies also have elucidated two broad categories of distress that are widely understood: one category is heart-mind problems (*manko samasya*) and the other category is brain-mind problems (*dimaagko samasya*) [32, 33]. The heart-mind is considered the organ of emotion and memories. For example, sadness, happiness, positive memories, and negative intrusive memories are seen as arising from the heart-mind. Heart-mind problems are considered commonplace and are not significantly stigmatized. Conversely, brain-mind problems refer to deficits in cognition, social behavior, morality, and rationality. Brain-mind problems, of which psychosis is an exemplar, are highly stigmatized. In addition to assessing detection of depression using the PHQ-9, this study also sought to evaluate the utility of eliciting heart-mind and brain-mind problems for detection of depression in primary care.

### Transcultural translation

A systematic approach for transcultural translation and adaption has been developed in Nepal [34], and has been used widely in cross-cultural mental health studies [35, 36]. The approach employs qualitative methods to optimize semantic, technical, content, criterion, and conceptual equivalence of a culturally-adapted tool compared to the original tool. The process employs four qualitative steps: (1) translation by bilingual speakers, (2) review by mental health professionals, (3) focus group discussions in which representatives of the patient population review each item, and (4) blind back-translation. The tool optimizes equivalence by evaluating four criteria: comprehensibility, acceptability, relevance, and completeness.

*Comprehensibility* is a measure of semantic equivalence and pertains to using appropriate idioms. If an item is deemed comprehensible by a focus group or individual, it is assumed understandable by a general audience in the specific cultural setting. *Acceptability and response set issues* reflect technical equivalence in how data are collected across cultures. If an item is deemed to have an acceptable response set, it suggests that respondents will rate items similarly to the original intention of the instrument. *Relevance* of items demonstrates content equivalence. Relevance is a measure of whether the item has locally significant meaning. For example, even though children may understand an item related to “watching television” or “playing video games,” the item may not be relevant in some LMIC settings where rural children may not have access to electricity and these leisure activities. *Completeness* combines semantic, criterion, and conceptual equivalence, thus capturing whether a question relates to the same concepts and ideas as the original item. Completeness accounts for cultural norms in relation to markers of psychopathology. For example, even though decreased sexual interest may be a comprehensible item (people understand the term) and relevant (sexual relations occur in the all of the world’s cultures), it may not be a marker of depression in a culture where it is not socially accepted for women to endorse interest in sex. Both depressed and non-depressed women would be equally likely to endorse low sexual interest in that population due to cultural norms [37]. The criterion of completeness can thus be employed regarding the construct to be measured; for example, does the item reflect the experience of depression.

This systematic approach to transcultural translation was used to adapt the PHQ-9. The PHQ-9 is a self-report screening tool for patients in various medical settings and was developed as a self-report based on the PRIME-MD [38]. The first draft of the Nepali tool was created by translators at TPO Nepal with extensive experience in translating mental health terminology between Nepali and English. Then mental health professionals including a psychologist and psychiatrist reviewed the tool. This was followed by a series of focus group discussions with laypersons in Chitwan, Nepal. The tool then underwent a blind back-translation and the research team reviewed the pre- and post-adaptation versions. Modifications were made as needed for the tool to meet the original conceptual objectives. Acceptability, relevance, comprehensibility, and completeness were reviewed at each step using the transcultural translation monitoring form [34]. The tool was also reviewed based on the association of items with feelings of sadness (Nepali, *manmaa dukha laagchha*) and impairment in daily functioning. Qualitative analyses were conducted using the pre-existing themes of acceptability, relevance, comprehensibility, and completeness. Two researchers reviewed transcripts.

### Participants for transcultural translation component

Participants for the focus group discussions were recruited from program communities in Chitwan. These participants were representative of the anticipated patient population. Focus group participants were selected according to gender and caste/ethnicity categorizations in Nepal in order to optimize feedback from diverse community beneficiaries. The distribution of caste/ethnicity was representative of the beneficiary population in rural southern Nepal based on local census figures [19].

### Validation

Validation was conducted by comparing the researcher-administrated Nepali PHQ-9 and a clinician administered structured interview which was administered by Nepali psychosocial counselors who received extensive training on the tool. Clinicians administered the depression module of the Composite International Diagnostic Interview (CIDI). The Nepali-language CIDI has been validated in Nepal, (AUC any disorder = 0.85, AUC depression = 0.97) [39]. The clinicians were Nepali psychosocial counselors. Their CIDI-based diagnoses were used to provide the “gold-standard” depression diagnosis for clinical validity. Utilizing psychosocial counselors, as opposed to clinical psychologists or psychiatrists, is based on a procedure previously developed by TPO Nepal for validating instruments in the context of limited availability of mental health experts [40]. Nepali psychosocial counselors represent the highest level of specialization below the expert level of psychologists and psychiatrists. Nepali psychosocial counselors are trained with a standardized 6-month curriculum including 400 h of classroom learning, 150 h of clinical supervision, 350 h of practice, and 10 h of personal therapy [41]. The psychosocial counselors selected for CIDI training in the current study had five or more years of clinical experience and at least 1 year in a supervisory or training role. The psychosocial counselors received a week of training in the Nepali CIDI including 6 hours of observed administration and review of scoring. An Australian psychologist with experience in conducting structured clinical interviews led the training. An expatriate psychologist and an expatriate psychiatrist, who are both fluent in Nepali, provided additional training and detailed review of videotaped CIDI interviews. Interviews were practiced until a rater achieved an intra-class correlation coefficient > 0.80. The final intra-class correlation coefficient for psychosocial counselor CIDI evaluations prior to the validation study was 0.93 (95 % confidence interval (CI), 0.90-0.98, single measures).

Patients first completed the PHQ-9 administered by a trained researcher. In addition to the PHQ-9, basic demographic information was collected from participants. They also were asked two questions related to local idioms of distress; if they had any heart-mind problems and if they had any brain-mind problems in the prior 2 weeks. The questions were open-ended and respondents could provide details if they chose to, or simply reply yes or no. Patients then participated in the CIDI structured interview with psychosocial counselors. Psychosocial counselors performing the CIDI were blind to the patient’s PHQ-9 score.

Analyses for the validation were conducted using descriptive statistics and comparisons of the results from the PHQ-9 or local idioms of distress with the CIDI structured clinical assessments. Diagnostic sensitivity and specificity, positive and negative predictive value (PPV and NPV), positive and negative likelihood ratios (LOR+ and LOR-respectively), and Youden’s Index (*J*) were calculated. Analyses were done for the entire sample in SPSS version 22.0 [42].

### Participants for validation component

For the validation study, patients were randomly selected from multiple primary care facilities in rural Chitwan. Patients older than 18 years of age were eligible as long as they were able to speak Nepali, could complete the consent process, and did not have any active health problems impairing hearing questions read to them.

### Ethical approval

Ethical approval was obtained from the Nepal Health Research Council. Data were collected in the period of January to April 2013. All participants completed a consent process in which they were read a consent form. Participants did not receive monetary compensation. Participants endorsing distress at the end of the interview or endorsing suicidal ideation were referred to mental health services, either counseling or evaluation by medical personnel. All participants approached for consent agreed to participate in the study; there were no refusals.

## Results

### Transcultural translation

The PHQ-9 underwent the above-mentioned four-step translation process. After completion of the translation and review by mental health professionals, the draft Nepali PHQ-9 was reviewed by four focus groups: two female groups, one male group, and one group with eight men and one woman (Table 1). One third of participants were illiterate, and one third had a primary school education. The age of respondents ranged from 18 to 80 years old.

**Table 1:** Demographics of Focus Groups

	Group No. 1	Group No. 2	Group No. 3	Group No. 4	Total (%)
Number of participants	11	10	8	9	38
Gender					
Male	0	10	0	8	18 (47 %)
Female	11	0	8	1	20 (53 %)
Caste/Ethnicity					
Brahman/Chhetri	4	9	0	1	14 (37 %)
Dalit	1	0	0	2	3 (8 %)
Janajati	6	1	8	6	21 (55 %)
Education					
Illiterate	3	3	2	5	13 (34 %)
Primary	5	4	3	2	14 (37 %)
Secondary or greater	3	3	3	2	11 (29 %)
Age, Mean (Range)	37 (24–60)	57 (19–80)	32 (21–45)	38 (20–60)	42 (19–80)

### Comprehensibility (semantic equivalence)

In focus group discussions, participants evaluated the terms used to describe each item (see Additional file 1 for final Nepali version and English back-translation). When terminology was difficult to understand, alternative terms and phrasing were developed. For example, in Item #2 the English idiom “feeling down” and the psychiatric term “depressed” were not comprehensible when translated directly into Nepali. Instead, language was developed referring to frustration (*dikka*), despair (*niraash)*, and feeling as if you are unable to do anything. Item #6 “feeling bad about yourself” and feeling that you “let yourself or your family down” was changed to blaming oneself and feeling unsuccessful, particularly in matters related to family. To address the impact on the family, the Nepali concept of *ijjat* was employed. *Ijjat* refers to social status and is associated with family standing. The Nepali idiom *ijjat gumaune*, which can be translated as wasting one’s family’s social status, was added. Participants of the focus groups easily understood these Nepali concepts and phrases. For Item #8 regarding psychomotor agitation, there is a Nepali idiom *chhatpatti,* which can be applied to both children and adults and refers hyperactivity and moving around too much. Respondents easily understood this, as well.

Another element of comprehensibility was the need to select examples that could be used which were culturally appropriate. For example, with regard to concentration impairment, examples of activities such as sorting rice, cooking vegetables, and cutting grass were introduced. Similarly, the term for suicide (*aatmahatyaa*) has limited understanding so we added common examples of self-harm and suicide including cutting one’s hands, taking poison, jumping from a high place, and banging one’s head.

### Acceptability and other response set issues (technical equivalence)

It is important to assure that items will not be stigmatizing or offensive. The only item that was contentious was suicidality. High-caste *Brahman/Chhetri* men stated that it was not appropriate, nor necessary, to ask about suicide: “You cannot ask about this because only *paagal* (mad, crazy) people have it,” stated a respondent in the high-caste men’s group, to which other participants in the group agreed. This reflects one cultural model of suicide in Nepal, one that is more common among males and those in positions of power. In contrast, both the women’s groups and the men’s group comprised of low caste/ethnic minority participants said that it was both acceptable and important to ask about suicide:

- “This is a serious problem for girls and women. They have fights with their husbands and then they try to do this.” - women’s group participant
- “The work burden of the house makes us [women] think about this sometimes because there is so much to do and no help from our husbands.” - women’s group participant
- “This is a serious problem during the period of studying for exams when there is so much tension.” - ethnic minority men’s group participant

Ultimately, the suicide item was retained because vulnerable groups identified the question as acceptable and relevant despite high-caste men saying that the question should not be included.

In the acceptability domain, the structure of the response set also was reviewed. Because the questions were being adapted to be asked by healthcare staff, the use of declarative phrases was difficult to understand. For example, reading only the phrase, “little interest or pleasure in doing things”, confused respondents about what they were being asked. Therefore, each PHQ-9 item was rephrased as a question. For example, Item 1 was rephrased as “In the past 2 weeks, compared to other people, how much do you feel that you don’t enjoy things, can’t enjoy yourself, can’t be happy, or don’t want to work?” The time-frame was repeated for every question to clarify that they were all referring to the same 2-week period. Other changes were made such as addressing ambiguous phrasing in the PHQ-9. On Item #8, the phrasing was clarified to address whether “others noticed or commented that you were moving slowly or too fast.”

The response options in the English PHQ-9 are “not at all”, “several days”, “more than half the days”, and “nearly every day”. In focus groups, participants reported difficulty with this because they felt they needed to know the exact number of days that a specific item occurred. This greatly prolonged response times while participants were trying recall each day in the past 2 weeks. Therefore, we modified the responses to more general responses of “not at all”, “sometimes”, “usually” and “always”. In addition, we added water glass response pictorial scale for endorsing the different levels (See Additional file 1). The water glass scale had previously been developed through a transcultural translation process with children in Nepal [40]. The adult participants in the current study reported that using the more general wording combined with water glass response scale was helpful to distinguish among different response options.

### Relevance and completeness (content, criterion, and conceptual equivalence)

In the focus groups, we discussed the degree to which the items have an association with *man dukhne* (heart-mind pain, an idiom referring to psychological distress typically in the form of sadness or despair) or “tension” (which is increasingly used in English by Nepali’s as way to describe stress and mild psychological distress). Participants reported, “We have a sleeping problem when we have tension,” and “sometime tension comes—maybe from any cause—then you cannot concentrate.” Participants also associated symptoms of the PHQ-9 with life events that cause stress, e.g., “Sometimes I am having a lack of interest in doing things when I have been having many quarrels with my family.”

The PHQ-9 was revised based on these results, then underwent blind back-translation. Minor modifications were made and the tool was finalized for use in the validation study.

### Validation

Participants were randomly recruited from rural health posts in Chitwan. Gender-stratified sampling was conducted to ensure balanced distribution. One hundred twenty-five participants were recruited (50 % female) (Table 2). The mean PHQ-9 score was 7.90 (standard error 0.50). Regarding idioms of distress, 95 participants (76 %) endorsed heart-mind problems and 12 (10 %) endorsed brain-mind problems.

**Table 2:** Demographics of validation study and depression status based on Composite International Diagnostic Interview (CIDI), *n* = 125

	Total Sample (*n* = 125)	CIDI Negative (*n* = 108)	CIDI Positive (*n* = 17)	Test-statistic	Significance
Gender					
Female	62 (50 %)	53 (49 %)	9 (53 %)	*Χ* ^2^ = 0.09	*p* = 0.77
Male	63 (50 %)	55 (51 %)	8 (47 %)		
Caste/Ethnicity					
Brahman/Chhetri	34 (27 %)	28 (26 %)	6 (35 %)	*Χ* ^2^ = 0.68	*p* = 0.71
Dalit	15 (12 %)	13 (12 %)	2 (12 %)		
Janajati	76 (61 %)	67 (62 %)	9 (53 %)		
Religion					
Hindu	106 (85 %)	93 (86 %)	13 (76 %)	*Χ* ^2^ = 5.90	*p* = 0.21
Christian	8 (6 %)	7 (6 %)	1 (6 %)		
Buddhist	7 (6 %)	4 (4 %)	3 (18 %)		
Muslim	3 (2 %)	3 (3 %)	0		
Other	1 (1 %)	1 (1 %)	0		
Education					
No formal education	44 (35 %)	34 (32 %)	10 (59 %)	*Χ* ^2^ = 5.40	*p* = 0.07
Primary	33 (26 %)	29 (27 %)	4 (23 %)		
Secondary or greater	48 (39 %)	45 (42 %)	3 (18 %)		
Psychiatric Care					
No history	121 (97 %)	105 (97 %)	16 (94 %)	*Χ* ^2^ = 0.46	*p* = 0.50
Any history	4 (3 %)	3 (3 %)	1 (6 %)		
Age, mean (95 % CI)	36.59 (Std Err = 1.52)	36.01 (1.58)	40.29 (4.90)	*t* = 0.83	*p* = 0.42
PHQ-9, mean (95 % CI)	7.90 (Std Err 0.497)	6.54 (0.41)	16.59 (1.14)	*t* = 8.84	*p* < .001
Heart-Mind Problems	95 (76 %)	79 (73 %)	16 (94 %)	*Χ* ^2^ = 3.54	*P* = .06
Brain-Mind Problems	12 (10 %)	4 (4 %)	8 (47 %)	*Χ* ^2^ = 31.81	*P* < .001

After completing the researcher-administered interview, participants underwent the structured diagnostic CIDI conducted by trained psychosocial counselors. Seventeen (14 %) of the 125 were positive according to the CIDI for a major depressive episode in the past 2 weeks. Of these, five had active suicidal ideation and were referred for care. The 17 CIDI-positive participants had less education and significantly greater PHQ-9 scores than CIDI negative participants did. The CIDI and PHQ-9 were compared identifying an area under the curve (AUC) of 0.94 (95 % CI 0.87—0.99). Psychometrics for different PHQ-9 cut-off scores are provided in Table 3. For a PHQ-9 score of 10 or greater, the sensitivity was 0.94 (95 % CI 0.73—0.99), specificity was 0.80 (95 % CI 0.71—0.86), PPV was 0.42 (95 % CI 0.27—0.59), and NPV was 0.99 (95 % CI 0.93—1.00), with a positive likelihood ratio of 4.62 (95 % CI 3.12—6.83), and negative likelihood ratio of 0.07 (95 % CI 0.01—0.47). Heart-mind problems had a sensitivity of 0.94 (95 % CI 0.69—1.00), specificity of 0.27 (95 % CI 0.19—0.36), PPV of 0.17 (95 % CI 0.10—0.26), and NPV of 0.97 (95 % CI 0.81—1.00). Brain-mind problems had low sensitivity for CIDI positive status (sensitivity = 0.47, 95 % CI 0.25—0.71).

**Table 3:** Psychometric properts of Patient Health Questionnaire (PHQ-9) for major depressive disorder (Composite International Diagnostic Interview), *n* = 125

	True Positive Cases	True Negative Cases	Sensitivity	Specificity	Positive Predictive Value	Negative Predictive Value	Positive Likelihood Ratio	Negative Likelihood Ratio	Diagnostic Odds Ratio	Youden’s Index (*J*)
PHQ -9 Cut Off Score										
≥ 7	17	59	1.00	0.55	0.26	1.00	2.20	0.00	n/a	0.55
≥ 8	17	67	1.00	0.62	0.29	1.00	2.63	0.00	n/a	0.62
≥ 9	16	75	0.94	0.69	0.33	0.99	3.08	0.08	36.36	0.63
≥ 10	16	86	0.94	0.80	0.42	0.99	4.62	0.07	62.55	0.74
≥ 11	15	90	0.88	0.83	0.45	0.98	5.29	0.14	37.50	0.71
≥ 12	15	94	0.88	0.87	0.52	0.98	6.81	0.14	50.36	0.75
≥ 13	14	97	0.82	0.90	0.56	0.97	8.09	0.20	41.15	0.72
≥ 14	12	98	0.71	0.91	0.55	0.95	7.62	0.32	23.52	0.62
≥ 15	10	103	0.59	0.95	0.67	0.94	12.71	0.43	29.43	0.54
Heart-mind (*man*) problems	16	29	0.94	0.27	0.17	0.97	1.29	0.22	5.87	0.21
Brain-mind (*dimaag*) problems	9	103	0.47	0.97	0.75	0.91	16.74	0.54	30.90	0.44
Algorithm	15	90	0.88	0.83	0.45	0.98	5.29	0.14	37.50	0.71

**Fig. 1:**
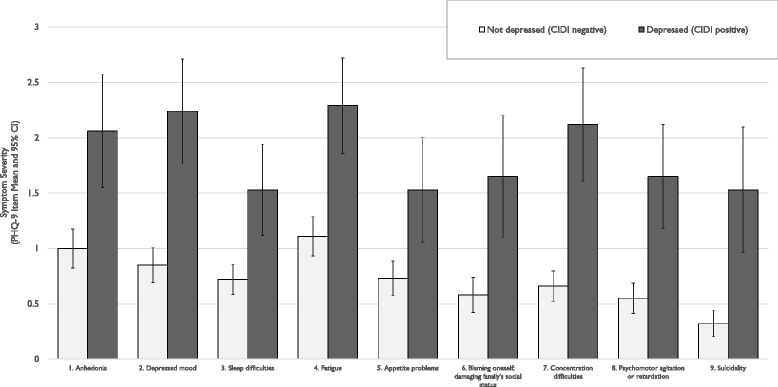
Patient Health Questionnaire (PHQ-9) item means and 95 % CI comparing non-depressed participants (Composite International Diagnostic Interview, CIDI negative), *n* = 108, and depressed participants (CIDI positive), *n* = 17). All comparison are significant, *p* < .02 after Bonferroni correction

Cronbach’s alpha for the tool was 0.84. There were fair item-total correlations in the range of .54 to .68 for eight of the items, with the exception of Item #5 (appetite problems) standing out as the lowest inter-item correlation at 0.37 (Table 4). In individual item-level analyses, all item means were significantly different when comparing non-depressed (CIDI negative) and depressed (CIDI positive) participants after Bonferroni-type corrections for multiple testing (Fig. 1), which demonstrates that CIDI positive participants as a group were more likely to endorse items #1 through #9 than CIDI negative participants were. The greatest positive likelihood ratios were observed for Item #6 “blaming oneself; damaging family status” (LOR+’ve = 1.89), Item #8 “psychomotor retardation or agitation” (LOR+’ve = 2.03), and Item #9 “suicidality” (LOR+’ve = 3.07).

**Table 4:** Item-Level Comparisons for Patient Health Questionnaire (PHQ-9) Nepali Items, *n* = 125

Item	Non-depressed (CIDI negative)		Depressed (CIDI positive)		T-test	Adjusted *p*-value*	Corrected Item-Total Correlation	Positive Likelihood Ratio
	Mean	Std. Dev.	Mean	Std. Dev				
1. Anhedonia	1.00	0.96	2.06	1.09	4.16	<.001	0.54	1.34
2. Depressed mood	0.85	0.84	2.24	0.97	6.18	<.001	0.68	1.49
3. Sleep difficulties	0.72	0.77	1.53	0.87	3.94	<.001	0.56	1.59
4. Fatigue	1.11	0.89	2.29	0.92	5.07	<.001	0.56	1.33
5. Appetite problems	0.73	0.87	1.53	1.00	3.44	0.01	0.37	1.62
6. Blaming oneself; damaging family’s social status	0.58	0.81	1.65	1.17	3.62	0.02	0.54	1.89
7. Concentration difficulties	0.66	0.71	2.12	1.05	5.52	<.001	0.68	1.72
8. Psychomotor agitation or retardation	0.55	0.76	1.65	0.99	5.24	<.001	0.55	2.03
9. Suicidality	0.32	0.59	1.53	1.18	4.14	0.01	0.57	3.07

* *p*-value corrected for 9 tests using Bonferroni-type corrections. Adjusted *p*-values are only presented for those items with significant unadjusted *p*-values

### Algorithm development

**Fig. 2:**
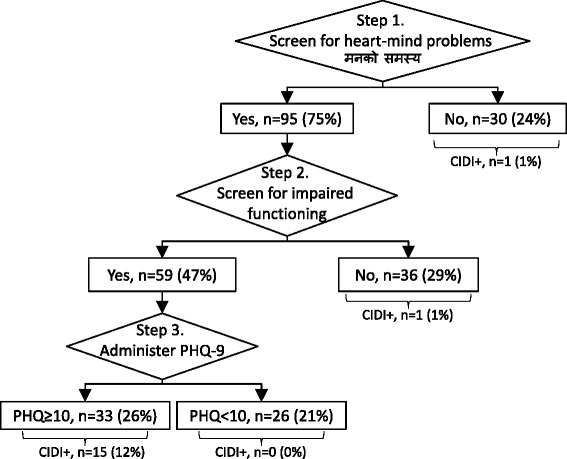
Algorithm for detection of depression in primary care in Nepal. Percentages refer to percent of total persons screened beginning at Step 1. Composite International Diagnostic Interview (CIDI+) refers to patients positive for major depressive disorder module with a 2-week time frame. Patient Health Questionnaire (PHQ-9) scores are using a ≥10 cut-off validated for rural Nepali populations in primary care

We then developed an algorithm for screening in primary care to optimize detection of depression while balancing time spent by health workers on a screening procedure in the context of setting with limited health personnel and resources (see tool in Additional file 1 in for stepwise screening). The algorithm employs three steps (Fig. 2):

Step 1. Screen patients for heart-mind problems
Step 2. Screen for impaired functioning due to heart-mind problems among patients positive for heart-mind problems
Step 3. Administer the full PHQ-9 to patients who screen positive on both heart-mind problems and function impairment due to heart-mind problems.

Based on the algorithm, the number of patients requiring PHQ-9 completion could be reduced by 50 %, and the number of false positives are reduced by 18 %. The algorithm’s cumulative psychometrics properties were sensitivity = 0.88 (95 % CI 0.62—0.98), specificity = 0.83 (95 % CI 0.75—0.90), PPV = 0.45 (95 % CI 0.29—0.63), NPV = 0.98 (95 % CI 0.92—0.99), positive likelihood ratio = 5.29 (95 % CI 3.36—8.35), and negative likelihood ratio = 0.14 (95 % CI 0.04—0.52). Original data are provided Additional file 2. 

## Discussion

A major barrier to scaling up mental health services in low resource settings throughout the world is the ability to detect mental illnesses, such as depression. It is only in combination with accurate detection that quality services can be provided. Detecting depression in primary care is aided by valid tools that can be feasibly administered given constraints such as low literacy among patient population and limited human resources available to conduct screening. We explored solutions to these constraints in Nepal by validating the PHQ-9 for primary care administration and developing an algorithm incorporating the assessment of local idioms. The culturally-adapted PHQ-9 uses lay Nepali terminology that allows ease of administration by literate persons without specialized mental health training, such as health auxiliary staff. The validation demonstrated psychometric properties comparable to HIC settings [13, 43].

We used transcultural translation procedures with qualitative methods to adapt the PHQ-9. English idioms, e.g., “feeling down”, and medical terminology were removed and replaced with comprehensible Nepali terms. Certain widely used Nepali idioms such as *chhatpatti* for hyperactivity and *ijjat gumaune* referring to damaging family social status made questions easily comprehensible for respondents. Specific examples were required for numerous questions, such as attention-requiring behaviors and types of suicide and self-harm. Items with these modifications displayed the highest positive likelihood ratios. For example, the *chhatpatti* modified psychomotor agitation item had an individual item positive likelihood ratio of 2.03, and the *ijjat gumaune* modified blaming oneself item was 1.89. Suicide, which included the list of examples, had the highest positive likelihood ratio of 3.07. In a similar study adapting the PHQ-9 and other instruments in Ghana, the authors found that adapted items had comparable or greater likelihood ratios and inter-item correlations when compared to non-modified items [14].

Suicide was the only item with questionable acceptability. People in positions of power (high caste men) dismissed suicide as only a problem of “crazy” people, whereas women and ethnic minorities strongly endorsed the need to assess suicide because of its problem in their communities. This highlights the need to get multiple stakeholders views due to wide intra-cultural variation in acceptability, comprehensibility, and relevance, especially for sensitive topics such as suicide [44]. Moreover, this also demonstrates the benefit of having all of the higher-power persons in one focus group separated from other participant groups so that others were able to share freely their reflections without feeling the need to ascribe to hegemonic norms.

Due to high illiteracy rates, a health worker must administer the screening. This required changing the structure to interrogatives in complete sentences, as has been done for postpartum screener adaptation in Ghana [14]. We found a similar need in prior work with children in Nepal, wherein the social bias associated with declarative sentences was reduced by using question phrasing, and the addition of pictorial response options was helpful [40]. Minimizing instrument design elements that contribute to social desirability bias in important in medical settings and among cultures with a strong emphasis on social hierarchies [45].

The validation of the PHQ-9 against the CIDI as a structured diagnostic interview produced results comparable to those found in high-income country primary care and other studies. A cut-off score of 10 produced psychometric properties (sensitivity = 0.94, specificity = 0.80, PPV = 0.42, LOR + = 4.62) comparable to findings with systematic reviews and meta-analyses of the PHQ-9 mostly representing high income countries: sensitivity = 0.77, specificity = 0.94, PPV = 0.59 [46]; sensitivity = 0.80, specificity = 0.92, LOR + =10.12 [12]. The psychometric properties are comparable to or better than those identified from PHQ-9 validation in other cross-cultural settings outside of HIC, e.g., Thailand: sensitivity = 0.84, specificity = 0.77, PPV = 0.21, LOR + =3.71 [47]; Malaysia: sensitivity = 0.87, specificity = 0.82, LOR + =4.8 [48]; South Africa: sensitivity = 51 %, specificity = 94 %, LOR + =7.78 [49]. The psychometric properties are comparable to or better than other instruments that have been validated in Nepal, such as the Beck Depression Inventory (BDI), sensitivity = 0.86, specificity = 0.87 [50], and the Hopkins Symptom Checklist (HSCL), sensitivity = 0.87, specificity = 0.60 [51]. However, it is important to note that the BDI and HSCL were validated with community samples and not in primary care settings as the current study was done. Other tools, such as the Center for Epidemiologic Studies Depression Scale (CES-D), have been used in Nepal but not validated against clinical diagnostic evaluations [52]. In this discussion, we chose to focus on a PHQ-9 cut-off score of 10 which optimizes sensitivity and has the highest diagnostic odds ratio (OR = 62.55). Depending on the intended use of the tool, a higher cut-off could be selected to optimize specificity.

High rates of false positives are seen as a limitation of the PHQ-9. In HIC settings, approximately four to six patients out of ten are falsely screened positive by ultra-short and short screening tools [11–13]. The Nepali PHQ-9 in this clinical sample similarly results in six out of ten positively screened patients being false positives. Although the result is comparable to HIC settings, the finding raises concerns about the benefit of screening and echoes controversies about screening’s utility [7].

One notable finding of the PHQ-9 was the low item-total correlation of appetite problems (Item #5) compared with the item-total correlation for other items. This is consistent with our findings for both the BDI among adults and the Depression Self Rating Scale (DSRS) among children [40, 53]. In both of these adult and child studies, the question regarding appetite changes and abdominal complaints performed very poorly. High rates of parasitic disease, gastrointestinal infections, and gastritis may contribute to prevalent appetite change complaints in Nepal and other low resource settings [53]. The poor performance of Item #5 may reflect a cultural emphasis on abdominal somatic complaints in Western cultural settings whereas other somatic complaints may be more effective for identifying depression in Nepal, such as head-based complaints (headaches – *thauko dukhne*, *kapal polne*), paresthesia (*jham-jhamaaune*, which is strongly associated with BDI scores, [54]), or alternative wording for abdominal complaints (such as *gyastrik*) [53]. Given that somatic complaints are the most common presenting complaint worldwide for common mental disorders [55, 56], incorporating culturally-relevant somatic idioms could improve accuracy of detection. Such modifications are supported by findings in other LMIC settings. For example, in Haiti, a Kreyòl Distress Idioms screener displayed better psychometric properties than transculturally translated Beck Depression and Anxiety Inventories [57]. Future studies could assess the psychometric properties of the PHQ-9 with alternative somatic complaints inserted for Item #5 or in the form of additional items.

We also compared the CIDI with idioms of distress related to heart-mind problems and brain-mind problems. Heart-mind problems captured nearly all CIDI cases. Only one CIDI depression positive participant did not endorse this idiom. However, heart-mind problems were much more prevalent than CIDI-positive depression, thus illustrating that this idiom—as with many cultural concepts of distress—is non-specific and should not be taken as synonymous with clinical depression [16]. Brain-mind problems did not overlap with CIDI depression similarly to heart-mind problems. This represents the different nature of brain-mind problems as reflecting cognitive, behavioral, social, and moral behaviors that do not have a predictable and consistent relationship with depression. Moreover, brain-mind problems are highly stigmatized, which may have influenced the low endorsement (10 % of the total sample compared to 75 % of the total sample for heart-mind problems) [32]. Heart-mind problems are an ideal screen for depression given the ability to capture the majority of cases and its non-stigmatizing social valence.

Of note, English idioms are increasingly used in Nepali, and the English term ‘tension’ was commonly reported by participants in our qualitative phase. ‘Tension’ has been reported in studies of psychological distress in Nepal [58]. ‘Tension’ has been used as a non-stigmatizing term to assess distress in India [59] and could be employed in future screening in Nepal. Idioms for “thinking too much” may also be effective to identify common mental disorders in Nepal and among other cultural groups throughout the world [60]. Development of tools that rely entirely upon local idioms and culturally salient indices of distress is another alternative for assessment; we have previously employed locally-developed tools successfully to identify mental health problems among former child soldiers and adult civilians in Nepal [21, 61].

### Application: algorithm for detecting depression in primary care

To improve clinical efficiency, we developed a stepped-algorithm employing, first, the assessment of a local idiom of distress (heart-mind problems) and, second, administering the PHQ-9. Only participants who answer yes to both of the idiom of distress question and a functional impairment question proceed to the PHQ-9. This procedure is advantageous because heart-mind problems are highly sensitive for CIDI positive depression status and they can be assessed with a single question. Screening for heart-mind problems eliminates one fourth of respondents from requiring further questions. Only one patient of the 17 was missed through heart-mind screening. In the next step, the PHQ-9 Item #10 can be used to assess function impairment related to heart-mind problems. When this is done, an additional third of the sample can be removed from the need for further screening, and only one CIDI positive depression participant was missed through functioning screening. Then, the PHQ-9 only needs to be administered to patients screening positive on heart-mind problems and related function impairment.

This algorithm reduces the total number of patients requiring PHQ-9 completion by 50 %, thus saving significant time and human resources in low-resource environments such as LMIC. The algorithm would contribute to less work for health auxiliaries conducting screening in a primary care setting. Without the algorithm, all persons presenting to a primary care setting would be given the full PHQ-9 to detect depression. Based on the algorithm, after asking the two idiom screening questions, only 59 of the 125 participants (47 % or primary care presenters) would need to complete the full PHQ-9. Thus, the amount of work for health auxiliary staff conducting screening would be reduced greatly.

This process also reduces the number of PHQ-9 false positives by 18 %. By reducing use of the PHQ-9 to only patients screening positive for the prior questions, the PPV is comparable to the screening all patients (PPV = 0.45 for algorithm vs. PPV = 0.42 for PHQ-9 administered to all) as is the NPV (1.00 for algorithm vs. 0.99 for screening all patients). The psychometric properties are comparable to using a higher cut-off of 11 on the PHQ-9, but have the added advantage of reducing the number of persons who require the full PHQ-9. Thus, detection and resources are optimized through an algorithm combining both cultural idioms of distress and the transculturally translated and validated PHQ-9. As with all screening, this does not equate with a diagnosis of major depressive disorder. Although the algorithm reduces false positives by 18 %, approximately half of the patients screening positive in this algorithm framework were false positives, which is comparable to rates in HIC.

A unique contribution of this algorithm is that it represents building upon prior approaches that have taken an either-or perspective when considering local idioms versus adapted psychiatric questionnaires. The strength is to combine both approaches. Local idioms and cultural concepts of distress are generally broad subjective categories with inter-individual differences in interpretation [17]. Local idioms are not synonymous with psychiatric categories and should not be used as a substitute for clinical assessment [16]. However, local idioms can be an efficient screen to then explore who may have a clinical disorder for which an evidence-based treatment is available. This algorithm may be particularly important in populations with chronic physical health problems. In Nepal, the rate of undiagnosed depression is 40 % among patients with diabetes [62] and 15 % among patients with hypertension [63].

In the context of the mhGAP initiative in Nepal and other countries, the benefits of using a tool such as the PHQ-9 in addition to the mhGAP-Intervention Guide needs further exploration. Does the addition of a screening tool to mhGAP guidelines optimize health system functioning and quality of care? An alternative to using the PHQ-9 could be incorporating screening for local idioms into the mhGAP assessment. Future studies are needed to evaluate patient outcomes, costs, and provider behavior using these different approaches. Currently, given the high rate of false positives and lack of data on quality of care outcomes, the *Nepal Mental Health Care Package* does not include recommendations for universal screening [27]. Of note, when services are present, the greatest potential harms of screening are pharmacological antidepressant treatments associated with suicidal thoughts and behavior and gastrointestinal bleeding; these harms are obviated in context where cognitive behavior therapy is the treatment of choice [9]. As PRIME research in Nepal explores the feasibility and effectiveness of pharmacological and/or psychological treatments in primary care, recommendations for screening may be modified [27].

A complimentary approach may be shifting from clinic-based screening to pro-active case finding at the community level. The recently developed Community-Informant Detection Tool (CIDT) is a procedure being piloted in Nepal in which community health workers and other community stakeholders are trained to use narrative-based pictorial case identification to identify and refer patients to primary care or other settings for evaluation [64]. The Nepali CIDT notably has better positive predictive value (PPV = 0.68) for detection of depression, compared to administration of the full PHQ-9 (PPV = 0.42). Therefore, the CIDT approach reduces health worker burden at the clinic level and reduces the number of false positives to one out three patients, which is better than HIC screener performance from the perspective of limiting false positives.

### Limitations

Limitations related to the study design and implementation must be taken into account when applying the findings to health services and clinical care. This study utilized a small sample size considered representative of the current PRIME initiative in Chitwan, Nepal. The psychometrics of the PHQ-9 may differ in other settings. For example, in Ethiopia the psychometric properties of screening tools for postnatal depression differed substantially between urban and rural settings [65]. Similarly, these psychometric properties are for a clinical primary care sample. Psychometrics would need to be adjusted for community-based studies, where the prevalence of depression is expected to be lower. In addition, we employed an alternative validation strategy in which experienced psychosocial workers were trained to administer the CIDI. This is an approach that we have previously employed successfully [40], and extensive training was provided with iterative evaluation of inter-rater reliability to optimize accurate use of the structured diagnostic tool.

An area requiring future study is sensitivity to change. As patients undergo treatment, does the PHQ-9 demonstrate treatment related responses, which has been shown when applying the PHQ-9 in some HIC settings [66]. Piloting is required to determine feasibility and accuracy when auxiliary health staff in a primary care clinic administer the algorithm. The present study does not include evaluation of test-retest reliability of the PHQ-9. Future studies are needed to establish this psychometric property.

## Conclusion

Detecting depression in low resource settings is crucial to assure that advances in availability of evidence-based care translate into delivery of care. Depression screening approaches from high-income countries using self-report questionnaires have limited application for populations with low literacy and lack of familiarity with completing such forms. We have shown that primary care workers can address this challenge by employing a stepped screening process with local idioms of distress followed by applying transculturally translated and clinically validated self-report questionnaires. Future research is needed to assess the implementation of similar algorithms in routine primary care in other cross-cultural low-resource settings. This research also points towards the need for more synthesis of standard psychiatric approaches with methods informed by cultural psychiatry and medical anthropology. Such innovations are required to address the high burden of disease due to depression globally.

## Additional files

Additional file 1: Nepal PHQ-9 Primary Care Depression Screening Tool. (PDF 483 kb) Additional file 2: Data file. (XLSX 45 kb)

## Abbreviations

AUC: area under the curve
BDI: beck depression inventory
CES-D: center for epidemiologic studies depression scale
CIDI: composite international diagnostic interview
CIDT: community informant detection tool
DFID: United Kingdom Department for International Development
DSRS: depression self rating scale
GCC: Grand Challenges Canada
HIC: high income countries
HSCL: Hopkins Symptom checklist
LMIC: low- and middle-income countries
LOR-: negative likelihood ratio
LOR+: positive likelihood ratio
mhGAP: Mental Health Gap Action Programme
NICE: United Kingdom’s National Institute for Health and Clinical Excellence
NIMH: National Institute of Mental Health
NPV: negative predictive value
PHQ-9: patient health questionnaire-9
PPV: positive predictive value
PRIME: Programme for Improving Mental Health Care
TPO Nepal: Transcultural Psychosocial Organization Nepal
WHO: World Health Organization
