# Supplementary material for: Detection of depression in low resource settings: validation of the Patient Health Questionnaire (PHQ-9) and cultural concepts of distress in Nepal
Source: BMC Psychiatry. 2016 Mar 8;16:58. doi: 10.1186/s12888-016-0768-y (PMC4782581; doi:10.1186/s12888-016-0768-y)
Supplement: Additional file 1: — Nepal PHQ-9 Primary Care Depression Screening Tool. (PDF 483 kb) [file 12888_2016_768_MOESM1_ESM.pdf]

प्राथमिक स्याहारमा उदासिनता पत्ता लगाउनकालागीप्रयोग गर्ने प्रश्नावली -नेपाल
Nepal Primary Care Depression Screening[1]

Introduction

अब हामी तपाईंलाई गएको २ हप्तामा एक दुई दिनमात्रै नभएर लगातार धेरै दिनसम्म मन दु:खी भईरहने, खिन्न लागि रहने वा नरमाईलो लागिरहने भएको थियो कि थिएन भन्ने वारेमा कुरा गर्छौ । Now we will be talking about your experience during the past two weeks where you have been continuously feeling, not only for a couple of days but continuously, sad in your heart-mind and unhappy.

Step 1. Heart-mind problem screening

तपाईलाई विगतको २ हप्तामा मनसँग सम्बन्धित कुनै समस्याहरु आएको थियो? जस्तै मनमा कुरा खेली रहने, मन दुखी भईरहने अथवा मनमा चिन्ता लागी रहने ? Have you had heart-mind problems in the past two weeks, for example thoughts playing in your heart-mind, sadness in your heart-mind, or worry in your heart-mind?

थियो<br>Yes	थिएन<br>No

यदी उत्तरदाताले प्रश्न नं १ मा थियो भन्ने उत्तर दिएमा दोस्रो चरणमा जानुहोस If the respondent answers yes to Question #1, proceed to step #2:

Step 2. Impairment due to heart-mind problem

गएको २ हप्तामा भर्खरै हामीले कुरा गरेको मन सँग सम्बन्धित समस्याहरुको कारणले तपाईलाई आफ्नो काम, आफ्नो साथै घरपरिवारको हेरचाह र अरू मानिसहरूसँगको तपाईको सम्बन्धमा कत्तिका असर पारेको थियो? During the past two weeks, have you experienced problems in your work, taking care of yourself and your family, or in your relationship with other people because of the problems that we talked about heart-mind problems?

थियो<br>Yes	थिएन<br>No

यदी उत्तरदाताले प्रश्न नं ३ माथि थियो भन्यो भने, तेस्रो चरणमा अघि बढनुहोस । If the respondent answers yes to Question #3, proceed to step #3:

[1] For additional information on administration and psychometric properties of this tool, please contact **Nagendra Luitel**, Research Department, Transcultural Psychosocial Organization Nepal, Kathmandu, Nepal, luitelnp@gmail.com. This tool was adapted and validated through the **Programme for Improving Mental Health Care (PRIME)** Research Consortiumwww.prime.uct.ac.za, funded by UK aid from the UK Government, however theviews expressed do not necessarily reflect the UK Government's official policies. The PHQ-9 was developed by Drs Robert L. Spitzer, Janet B.W. Williams, and Kurt Kroenke.

Step 3. Patient Health Questionnaire (PHQ-9)

	Nepali questions with English back-translation	कत्ति पनि भएन Not at all	कहिलेकाहीं भयो Sometimes	धेरै जसो भयो Usually	सधै जसो भयो Always
१.	गएको २ हप्तामा तपाईं अन्य व्यक्तिहरू जति रमाउँछन्, त्यत्तिको रमाउन नसक्ने/खुसीहुन नसक्ने वा काम गर्न मन नलाग्ने कत्तिको भएको थियो ? During the past two weeks, compared to other people, how much have felt that you are not able to be happy or do not enjoy doing work/activities?	०	१	२	३
२.	गएको २ हप्तामा तपाईलाई, दिक्क लाग्ने, निरास हुने वा केहीगर्न सक्दिन जस्तो लाग्ने कत्तिको भएको थियो ? During the past two weeks, how much have you felt frustrated, despairing,orincapable of doing anything?	०	१	२	३
३.	गएको २ हप्तामा तपाई राम्ररी सुत्न नसक्ने वा निन्द्रा मस्त नआउने वा अघिपछि भन्दा धेरै सुत्ने वा धेरै निन्द्रा लाग्ने कत्तिको भएको थियो ? During the past two weeks, how much have you had problems with your sleep, such as not being able to sleep properly and peacefully, or feeling sleepier than before?	०	१	२	३
४.	गएको २ हप्तामा तपाईंलाई थकान महशुस हुने, शक्ति वा तागत् कम हुने कत्तिको महशुस भएको थियो ? During the past two weeks, how much have you felt tired and lacking energy?	०	१	२	३
५.	गएको २ हप्तामा तपाईंलाई खानाखान मन नलाग्ने वा रुचि नहुने वा अघिपछि भन्दा धेरै खान मन लाग्ने कत्तिको भएको थियो ? During the past two weeks, how much have you lost your appetite or experienced increased appetite?	०	१	२	३
६.	गएको २ हप्तामा तपाईं आफूले आफूलाई दोषी ठान्ने वा आफ्नो कारणले गर्दा आफू असफल भएको वा परिवार तल परेको कत्तिको महशुस गर्नु भएको थियो ? (जस्तै:आफ्नो कारणले गर्दा समुदायमा आफ्नो तथा आफ्नो परिवारको ईज्जत गुमेको महशुस गर्ने) During the past two weeks, how much have you blamed yourself for something or felt that you have let you and your family down? (For example, because of you, you and your family have lost respect in the society.)	०	१	२	३
७.	गएको २ हप्तामा तपाईंलाई ध्यान केन्द्रित गर्न वा एक चित्त हुन वा एक सुरले काम गर्न गाह्रो हुने कत्तिको भएको थियो ?(जस्तै: पत्रपत्रिका पढ्न वा टी.भी. हेर्न, चामल केलाउन, पात गाँस्न, तरकारी पकाउन, घाँस काट्न, ध्यान दिएर कुनै पनि काम गर्न आदि) During the past two weeks, how much have you been having difficulty being able to focus or concentrate? (For example, not being able to concentrate while watching TV, reading newspaper, cleaning rice, cooking, cutting grass, or working.)	०	१	२	३
८.	गएको २ हप्तामा अरू व्यक्तिले पनि याद गर्ने गरी तपाईं बिस्तारै बोल्ने वाहिड्ने गर्नुभएको अथवा छट्पट्टीभएर चाहिने भन्दा बढी हिंडडुल गर्ने कत्तिको गर्नुभएको थियो ? During the past two weeks, how much have people commented that you have been talking very softly, walking slowly, moving around needlessly, or acting restless?	०	१	२	३
९.	गएको २ हप्तामातपाईंलाई मरौं मरौं जस्तो लाग्ने, आफ्नो ज्यान आफैं लिने वा आत्महत्या गर्ने खालका विचारहरू कत्तिको आयो ? (जस्तै: हातकाट्ने, विष सेवनगर्ने, हामफाल्ने, भित्तामाटाउको ठोक्काउने आदि) During the past two weeks, how much have you had the feeling of hurting yourself, dying, or commiting suicide? (For example, cutting your hands, taking poison, jumping from somewhere, hitting your head against the wall.)	०	१	२	३

जम्मा अङ्क [Total Score, PHQ-9, #1-9]: __________

Water Glasses Pictorial Response Scale

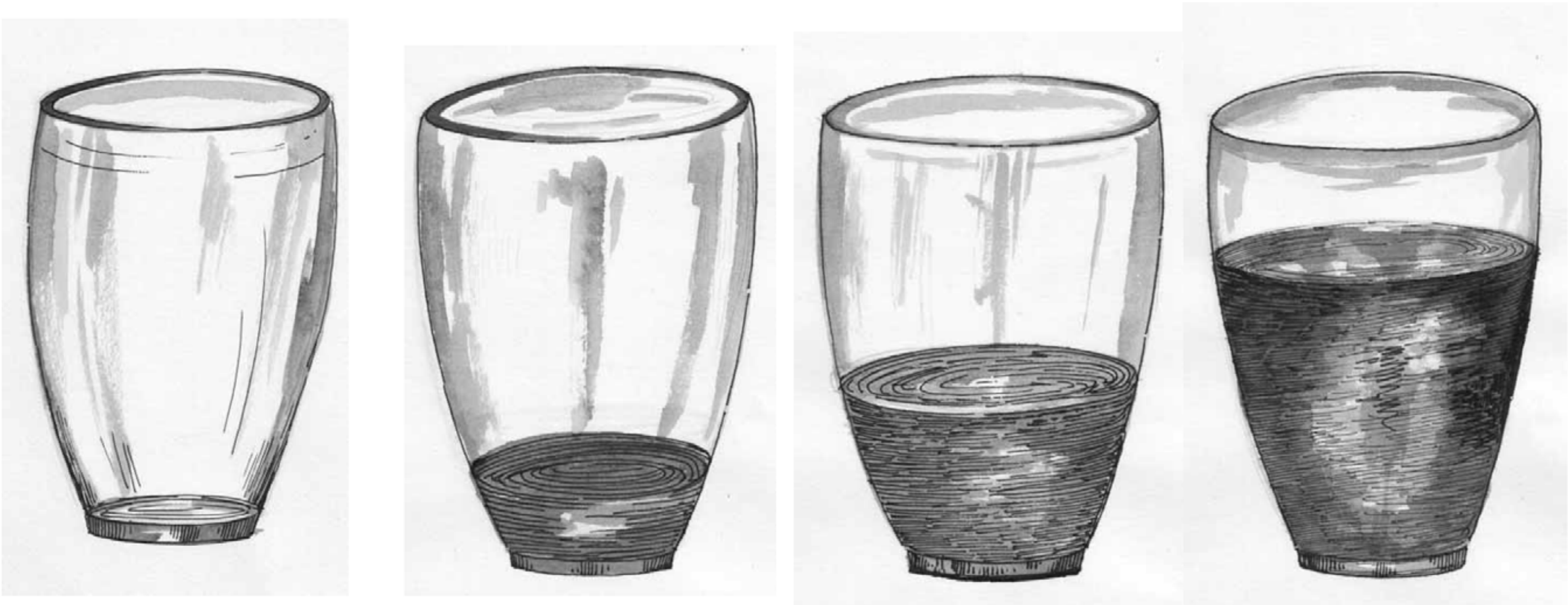

कत्ति पनि भएन<br>Not at all	कहिलेकाहीं भयो<br>Sometimes	धेरै जसो भयो<br>Usually	सधै जसो भयो<br>Always
०	१	२	३
